# Building Oncofertility Core Competency in Developing Countries: Experience From Egypt, Tunisia, Brazil, Peru, and Panama

**DOI:** 10.1200/JGO.17.00121

**Published:** 2018-02-07

**Authors:** Mahmoud Salama, Lauren Ataman, Tamer Taha, Osama Azmy, Marouen Braham, Fatma Douik, Mohamed Khrouf, Jhenifer Kliemchen Rodrigues, Fernando M. Reis, Flor Sánchez, Sergio Romero, Mario Vega, Teresa K. Woodruff

**Affiliations:** ^1^ National Research Center, Cairo, Egypt; ^2^ Northwestern University, Chicago, IL; ^3^ Aziza Othmana Hospital of Tunis, Tunisia; ^4^ FERTILLA, Clinique la Rose, Tunis, Tunisia; ^5^ In Vitro Consultoria–Research and Development/Clinical Embriology, Gerais, Brazil; ^6^ Universidade Federal de Minas Gerais, Belo Horizonte, Minas Gerais, Brazil; ^7^ Centro de Estudiose Investigaciones en Biología y Medicina Reproductiva, Lima, Peru; ^8^ Consultorios Hospital Punta Pacific, Panama City, Panama

## Abstract

**Purpose:**

Little is known about oncofertility practice in developing countries that usually suffer from a shortage of health services, especially those related to cancer care.

**Materials and Methods:**

To learn more about oncofertility practice in developing countries, we generated a survey to explore the barriers and opportunities associated with oncofertility practice in five developing countries from Africa and Latin America within our Oncofertility Consortium Global Partners Network. Responses from Egypt, Tunisia, Brazil, Peru, and Panama were collected, reviewed, and discussed.

**Results:**

Common barriers were identified by each country, including financial barriers (lack of insurance coverage and high out-of-pocket costs for patients), lack of awareness among providers and patients, cultural and religious constraints, and lack of funding to help to support oncofertility programs.

**Conclusion:**

Despite barriers to care, many opportunities exist to grow the field of oncofertility in these five developing countries. It is important to continue to engage stakeholders in developing countries and use powerful networks in the United States and other developed countries to aid in the acceptance of oncofertility on a global level.

## INTRODUCTION

Because of advances in cancer diagnosis and treatment, the overall survival rates in most young women and men with cancer have significantly increased over the past four decades.^1-3^ Consequently, the topic of how to prevent the chemotherapy- and radiotherapy-induced gonadotoxicity and subsequent fertility loss has gained attention. Oncofertility is a new interdisciplinary field at the intersection of oncology and reproductive medicine that expands fertility options for young cancer survivors.^4-8^ Throughout the past decade, international guidelines were published about oncofertility practice in developed countries.^9-11^ However, little is known about oncofertility practice in developing countries that usually suffer from a shortage of health services, especially those related to cancer care. In this study, we investigated oncofertility practice in developing countries and explore the unique barriers and opportunities for growth and expansion.

## MATERIALS AND METHODS

A pilot survey was generated after the panel session Resource Barriers to Oncofertility in Developing Countries: Challenges and Opportunities at the 10th Annual Oncofertility Conference, Feinberg School of Medicine, Northwestern University, Chicago, Illinois, November 1 to 3, 2016. To explore the various barriers and opportunities of oncofertility practice in developing countries, the survey questions were sent by e-mail to five centers from Africa and Latin America within the Oncofertility Consortium Global Partners Network (OCGPN).^12^ The surveyed centers from Egypt, Tunisia, Brazil, Peru, and Panama are listed in Table 1. The survey questions were grouped into six categories: country profile, cancer care, fertility treatments, fertility preservation treatments, barriers to oncofertility, and opportunities of oncofertility (Table 2). Responses from the surveyed centers were collected, reviewed, and discussed.

**TABLE 1:**
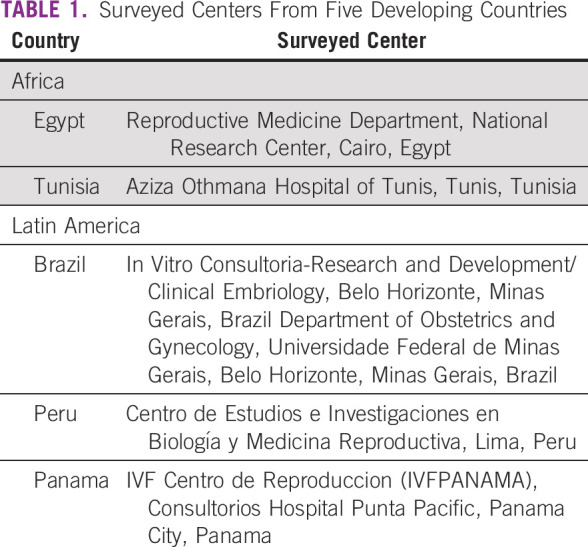
Surveyed Centers From Five Developing Countries

**TABLE 2:**
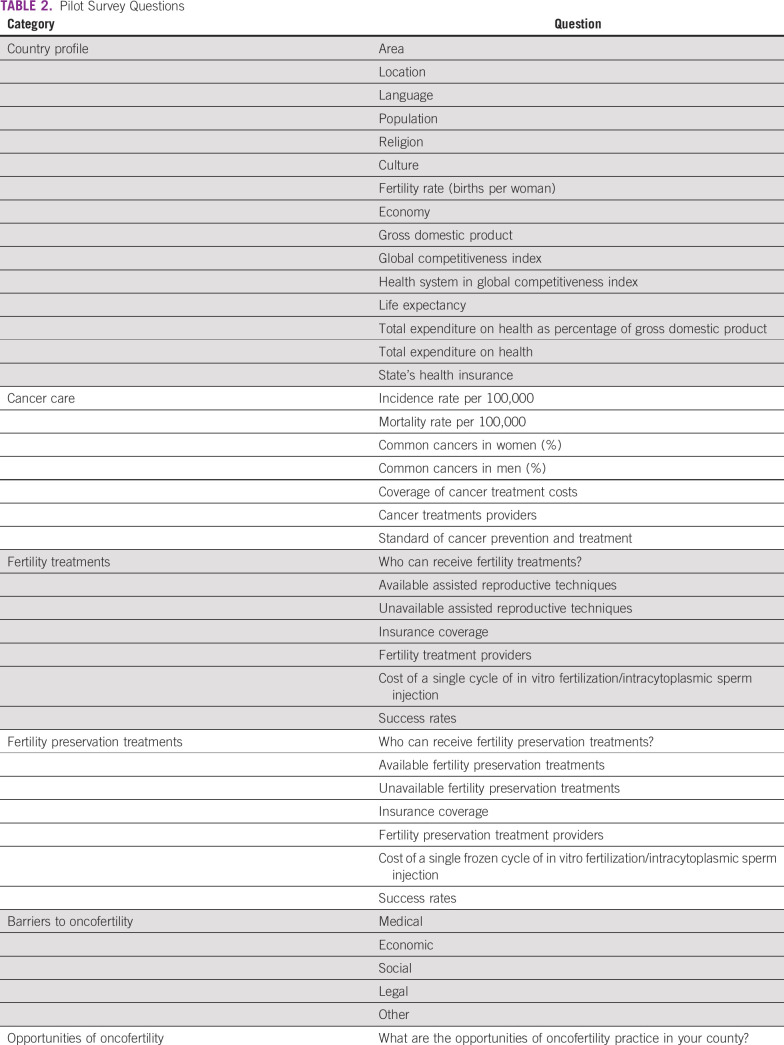
Pilot Survey Questions

## RESULTS

All surveyed centers from the five developing countries (Egypt, Tunisia, Brazil, Peru, and Panama) responded to all questions. Responses are listed in detail in Tables 3 to 8 (developing country profile 2015/2016, cancer care, fertility treatments, fertility preservation treatments, barriers to oncofertility, and opportunities of oncofertility, respectively).

**TABLE 3:**
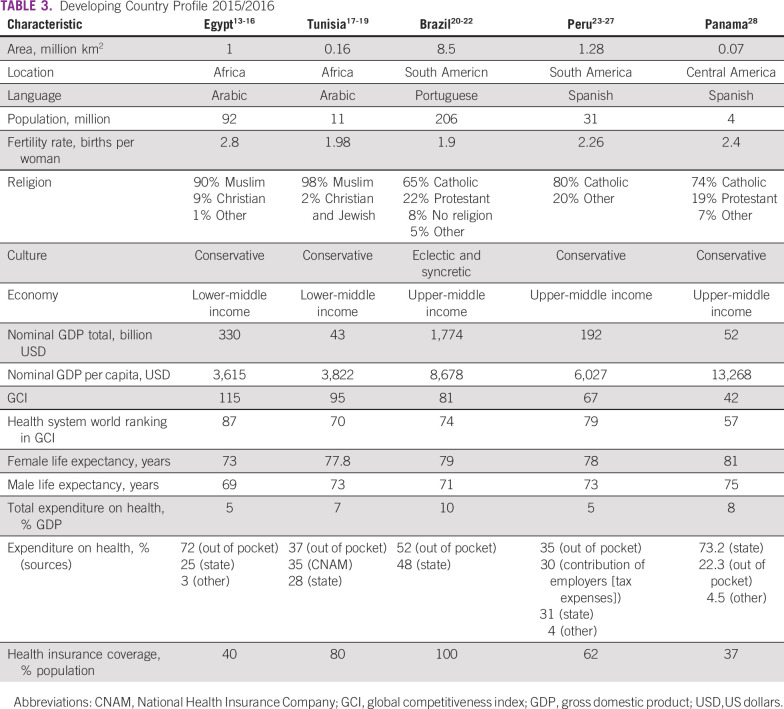
Developing Country Profile 2015/2016

**TABLE 4:**
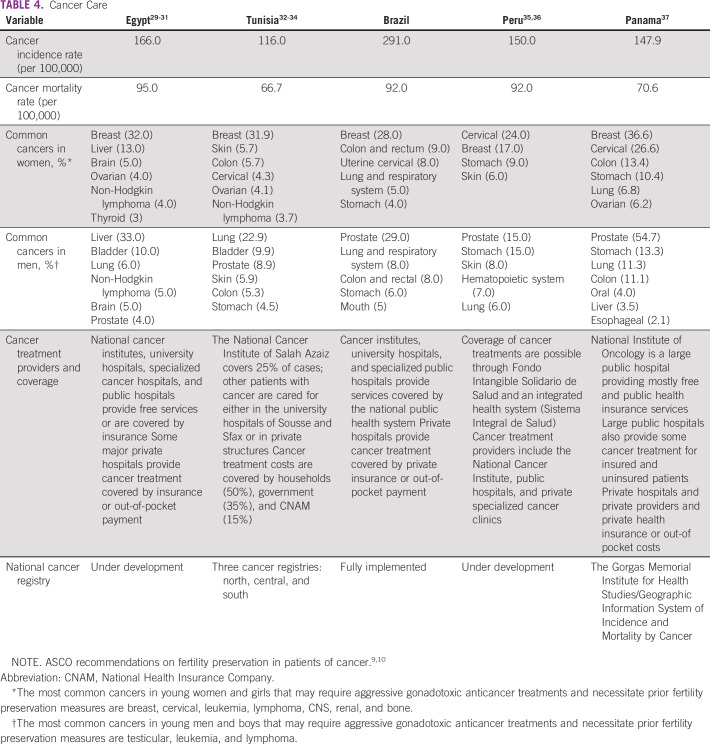
Cancer Care

**TABLE 5:**
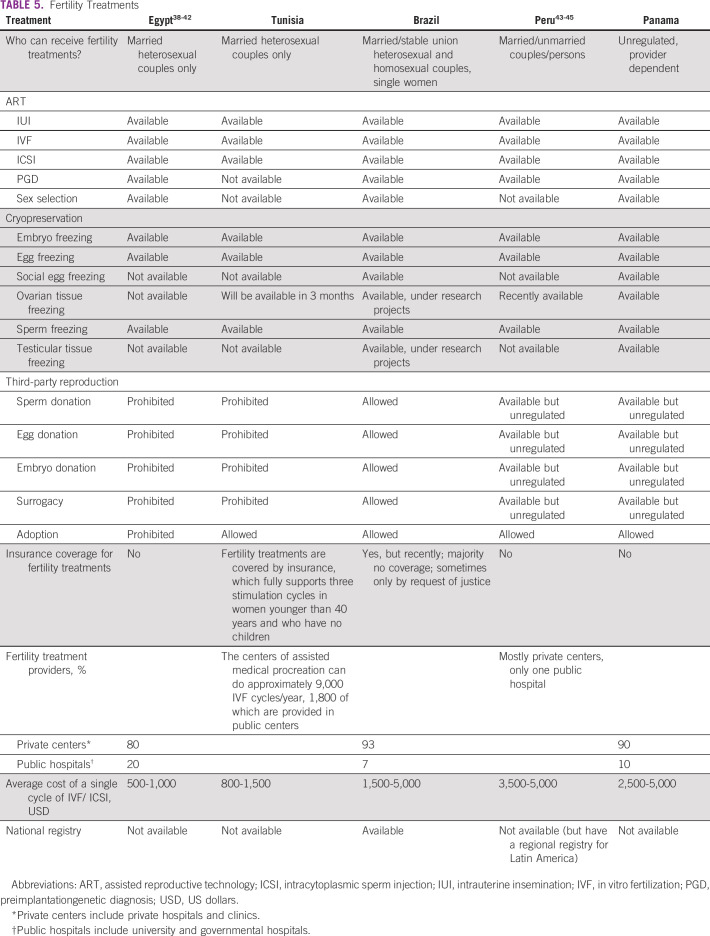
Fertility Treatments

**TABLE 6:**
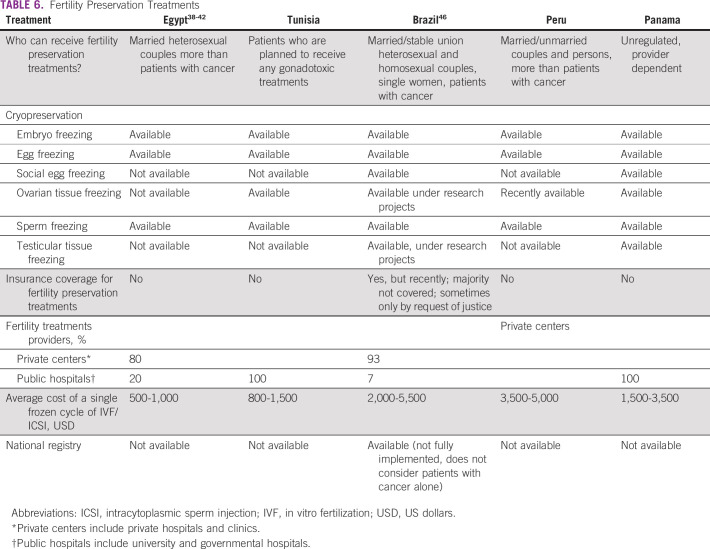
Fertility Preservation Treatments

**TABLE 7:**
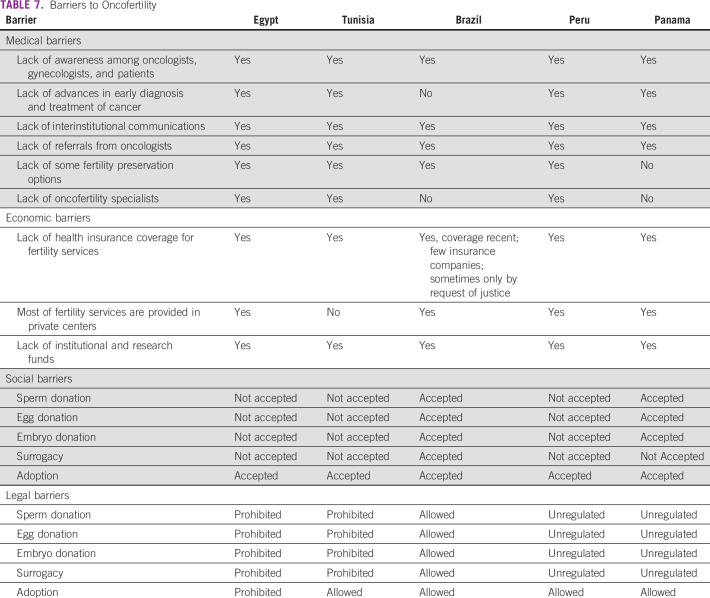
Barriers to Oncofertility

**TABLE 8:**
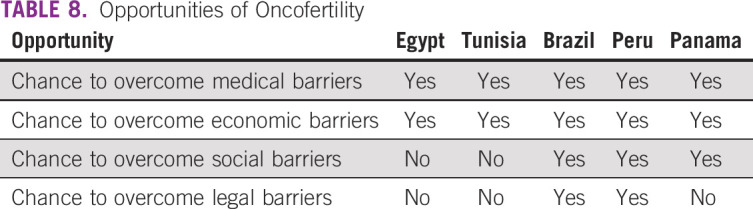
Opportunities of Oncofertility

## DISCUSSION

As survival rates continue to rise, the need for oncofertility services in developing countries has become increasingly apparent. Although the mortality rate remains relatively higher in developing countries than in developed ones, quality of life matters for all patients, including future fertility of adult cancer survivors. Because approximately 50% of cancer in developing countries occurs in individuals younger than 65 years,many patients are of reproductive age, and their reproductive health, including endocrine health, should be considered before treatment starts. However, with limited resources, including significant financial burdens to patients and their families such as high out-of-pocket expenses and limited insurance coverage, oncofertility services are not viewed as a necessity and are disproportionally available to affluent patients with the means to pay for these additional services. Furthermore, many providers and patients are focused on eliminating cancer and do not consider that fertility can be an important quality-of-life concern later on.

Respondents identified a number of barriers to oncofertility care in their countries. Common barriers are a lack of awareness among oncologists, lack of funds, high costs, and cultural and religious constraints that result in negative attitudes toward assisted reproduction technology and fertility preservation and oncofertility services. This general lack of awareness among providers may result in a reluctance to accept new technologies and practice.

Although survey respondents identified a number of barriers to care, these barriers can be positively viewed as opportunities for expansion, growth, and development. To facilitate collaborations and reduce duplicative efforts, the OCGPN was formed in 2012.^8^ The OCGPN works with reproductive specialists from 33 countries around the globe in an effort to better serve children, adolescents, young adults, and adults with cancer and other fertility-threatening diseases. It acts as an organizing center and fosters interaction among groups that can share resources, methodologies, and other experiences in the field. The establishment of a strong global network not only drives the collaborative nature of the consortium but also helps global partners to build their own programs and fertility preservation networks, as with the Brazilian Oncofertility Consortium, the Peruvian Oncofertility Network, and the Latin American Oncofertility Network. These networks were born out of the work of the OCGPN and are now models of success for future networks.

The OCGPN assists these developing countries as their programs develop and grow. All the survey respondents are current members of the consortium (Table 1), and members from both Brazil and Tunisia have adapted existing oncofertility materials to their native languages for their communities. For example, Brazil, one of the first countries to join the OCGPN, translated materials from Northwestern University’s Save My Fertility online fertility preservation toolkit^47^ to Portuguese so that their providers receive information in the native language. Furthermore, the group published an oncofertility textbook in Portuguese: *Preservação da Fertilidade: Uma Nova Fronteira Entre Medicina Reprodutiva e Oncologia*.^48^ This book is intended for health professionals to increase their understanding of fertility preservation in patients with cancer. Members from Aziza Othmana Hospital of Tunis, Tunisia, also translated Save My Fertility materials to French. These translation projects engage partners in consortium-wide activities, but more importantly, they bring utility to providers, patients, other health professionals in their home countries. Members of the OCGPN have hosted a number of conferences, meetings, and seminars in their home countries to educate their communities about oncofertility and fertility preservation in patients with cancer. Furthermore, the group has worked collectively on three publications that assist developing programs with justifying their program with local governmental or clinical governing bodies and with increasing awareness about oncofertility throughout their home institutions, countries, and regions.^8,^^49,50^ The goal is to reduce duplicative efforts and ease the burden of setting up an oncofertility practice in a country that may have limited resources.

Collaborations are imperative to the success of oncofertility centers in developing countries. However, networking is only one of the characteristics of a successful program. Persistence is key. With limited resources and lack of institutional support, oncofertility advocates easily could become discouraged with the process of setting up their own practice. These challenges are faced even in developed nations, and many people in the oncofertility community experience similar barriers. Even in a challenging environment, oncofertility champions must continue their work to increase awareness and advocate for increased access to fertility preservation services for cancer survivors.

Success is not defined by 100% of patients with cancer pursuing oncofertility services; success is defined simply as increased awareness among both patients and providers. Even in the United States and other developed nations, not all patients choose to pursue fertility preservation options, but the goal of the oncofertility community is to ensure that these conversations take place and that providers are empowered to navigate the complex fertility issues patients with cancer face.

This study had some limitations, including a lack of data on pediatric and adolescent and young adult cancer incidence in these countries. This patient population and their parents, guardians, and providers should be aware of the effects of cancer and cancer treatments on future fertility, reproductive health, and quality of life throughout survivorship. In the future, a similar study could examine the state of pediatric and adolescent and young adult oncofertility services in developing countries and implement strategies for increasing awareness.

Even with structural and financial limitations, there are many opportunities to expand oncofertility in developing countries. An increase of awareness about available fertility preservation options is the collective goal of the community. Continued tenacity, network building, and advocacy can accomplish this goal. The use of the services offered by the existing OCGPN will reduce duplicative efforts in developing countries. As a consequence of the unmet needs identified by survey respondents, the OCGPNC will use this survey as a baseline for developing countries to evaluate their own programs within the context of their country profile. This exercise will not only force sites to provide thorough self-evaluations, but also help them to identify shortcomings and opportunities for future development and success.

In conclusion, common barriers were identified by each country that responded to this survey. These barriers were lack of insurance coverage and high out-of-pocket costs for patients, lack of awareness among providers and patients, cultural and religious constraints, and lack of funding to help to support oncofertility programs. Despite these barriers, many opportunities exist to grow the field of oncofertility in these five developing countries. Continuing to engage stakeholders in developing countries and the use of powerful networks in the United States and other developed countries will aid in the acceptance of oncofertility on a global level.
